# Dose-dependent skeletal deficits due to varied reductions in mechanical loading in rats

**DOI:** 10.1038/s41526-020-0105-0

**Published:** 2020-05-18

**Authors:** Frank C. Ko, Marie Mortreux, Daniela Riveros, Janice A. Nagy, Seward B. Rutkove, Mary L. Bouxsein

**Affiliations:** 1 Center for Advanced Orthopaedic Studies, Beth Israel Deaconess Medical Center, Boston, MA USA; 2 Department of Neurology, Harvard Medical School, Boston, MA USA; 3 Department of Neurology, Beth Israel Deaconess Medical Center, Boston, MA USA; 4 Department of Orthopaedic Surgery, Harvard Medical School, Boston, MA USA

**Keywords:** Experimental models of disease, Preclinical research

## Abstract

Reduced skeletal loading leads to marked bone loss. Animal models of hindlimb suspension are widely used to assess alterations in skeleton during the course of complete unloading. More recently, the effects of partial unloading on the musculoskeletal system have been interrogated in mice and rats, revealing dose-dependent effects of partial weight bearing (PWB) on the skeleton and skeletal muscle. Here, we extended these studies to determine the structural and functional skeletal alterations in 14-week-old male Wister rats exposed to 20%, 40%, 70%, or 100% of body weight for 1, 2, or 4 weeks (*n* = 11–12/group). Using in vivo pQCT, we found that trabecular bone density at the proximal tibia declined in proportion to the degree of unloading and continued progressively with time, without evidence of a plateau by 4 weeks. Ex vivo measurements of trabecular microarchitecture in the distal femur by microcomputed tomography revealed deficits in bone volume fraction, 2 and 4 weeks after unloading. Histologic analyses of trabecular bone in the distal femur revealed the decreased osteoblast number and mineralizing surface in unloaded rats. Three-point bending of the femoral diaphysis indicated modest or no reductions in femoral stiffness and estimated modulus due to PWB. Our results suggest that this rat model of PWB leads to trabecular bone deterioration that is progressive and generally proportional to the degree of PWB, with minimal effects on cortical bone.

## Introduction

Reduction in mechanical loading occurs in many settings, including cast immobilization, extended bed rest, neuromuscular disorders, spinal cord injury, and spaceflight. Though dependent on the duration and extent of unloading, these reductions in mechanical loading lead to marked skeletal fragility, with reported loss of bone mineral density (BMD) ranging from 10 to 40%^1–5^. Thus, there is a need to develop strategies to mitigate bone loss associated with reduction in mechanical loading. Such strategies would be best informed by a better understanding of the nature of disuse-induced bone loss.

Animal models of reduced mechanical loading have been used to characterize bone loss and evaluate interventions designed to prevent the deleterious effects of unloading. These models include hindlimb unloading, cast immobilization, botulinum toxin administration, and nerve injury^6–10^. While effective in reflecting the musculoskeletal changes that occur with complete immobilization or unloading, these models are limited in their ability to assess musculoskeletal changes due to partial reductions in mechanical loading that occur in many situations. For example, there is an increased risk of fracture associated with muscular dystrophy, cerebral palsy, and stroke^11–13^, where patients are partially loaded. Furthermore, an animal model that could mimic the musculoskeletal effects of fractional gravity, such as that experienced on the Moon or Mars, could provide insights useful for planning future space missions.

To address this gap in knowledge, we had previously developed a partial weight-bearing (PWB) mouse model, and demonstrated the degree of skeletal deterioration to be closely related to the magnitude of unloading^14,15^. We recently expanded this PWB model to rats, which again showed dose-dependent loss of bone and muscle by varied reductions in weight bearing^16–19^. In the current study, we aimed to extend this work with a comprehensive evaluation of the time course of changes in bone density, microarchitecture, and strength following exposure to varied weight-bearing levels in adult rats. We also incorporated histomorphometric measurements to delineate the effects of PWB on bone metabolism at the cellular level. We hypothesized that partial reductions in mechanical loading will lead to proportional, progressive deteriorations in bone density, microarchitecture, and strength driven by increased bone resorption and reduced bone formation.

## Results

**Fig. 1:**
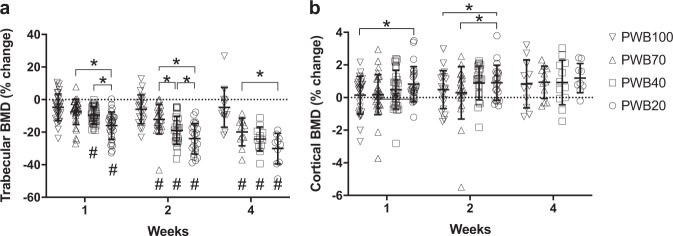
Peripheral quantitative computed tomography assessment of proximal tibial metaphysis. Dose-dependent reduction in proximal tibial trabecular bone density **a** and moderate increase in cortical bone density **b**. Data were analyzed by linear mixed model (fixed variable: PWB, experimental duration, and their interaction; repeated variable: experimental duration) followed by pairwise comparisons with Bonferroni correction. Data represent mean ± SD. ^#^*p* < 0.05 vs PWB100 within the same time point; *p < 0.05 vs other groups within the same time point.

In vivo longitudinal assessment of tibial metaphyseal trabecular bone density showed both load- and time-dependent effects of PWB (Fig. 1). The dose-dependent response to unloading was evident as early as 1 week after unloading (test for trend, *p* < 0.0001). In particular, trabecular volumetric BMD (vBMD) declined by 18% in PWB20 (*p* < 0.001 vs baseline), 9% in PWB40 (*p* < 0.001 vs baseline), 9% in PWB70 (*p* < 0.001 vs baseline), and 5% in PWB100 (*p* < 0.01 vs baseline). Whereas trabecular vBMD remained stable in the PWB100 group over time, longer exposure to unloading led to progressive declines in trabecular vBMD in all groups, with maintenance of the dose-dependent response at all subsequent timepoints (i.e., 2 and 4 weeks; test for trend, *p* < 0.0001). Notably, we did not observe any plateau in the decline in trabecular vBMD. By 4 weeks after unloading, trabecular vBMD had declined by 34%, 24%, and 23% from baseline in the PWB20, PWB40, and PWB70 groups, respectively.

Unlike trabecular bone density, alterations in cortical bone density at the mid-diaphysis were moderate in most groups. In the PWB20 group at the 1-week (0.8%, *p* < 0.001 vs baseline), 2-week (0.9%, *p* < 0.001 vs baseline), and 4-week (1.2%, *p* = 0.008 vs baseline) time points. Also a slight increase in cortical bone density was observed in the PWB40 group at the 2-week (0.9%, *p* = 0.035 vs baseline) and 4-week (0.9%, *p* = 0.016 vs baseline) time points.

### Bone microarchitecture

**Fig. 2:**
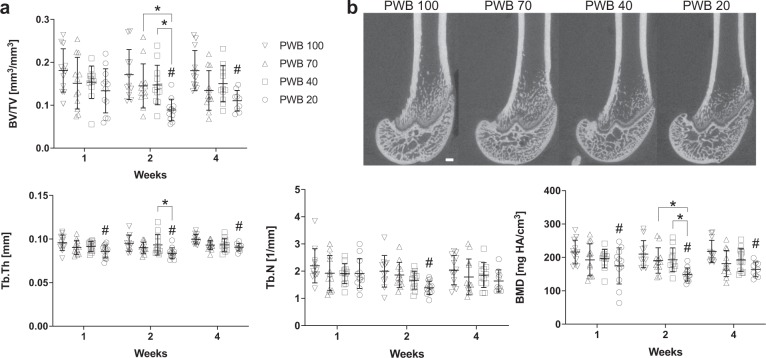
Microcomputed tomography assessment of distal femoral metaphysis. Dose-dependent reduction in distal femoral trabecular bone properties **a**. Representative microCT images of distal femur at 4 weeks **b**. BV/TV bone volume/total volume, Tb.Th trabecular thickness, Tb.N trabecular number, BMD bone mineral density; scale bar = 500 μm; data were analyzed by two-way ANOVA followed by Tukey’s post hoc tests. Data represent mean ± SD. ^#^*p* < 0.05 vs PWB100 within the same time point; *p < 0.05 vs other groups within the same time point.

MicroCT imaging revealed that trabecular microarchitecture in the distal femoral metaphysis also deteriorated according to the PWB level (Fig. 2a). For example, in the PWB20 group trabecular bone volume/total volume (Tb.BV/TV) as 48% and 39% lower compared to PWB100 after 2 and 4 weeks of unloading, respectively. Further, Tb.BV/TV in PWB20 was ~40% lower than that of PWB40 and PWB70 at 2 weeks. This decrease in Tb.BV/TV was due to both thinning of trabeculae and reduced trabecular number (Fig. 2b). Cortical bone microarchitecture at the femoral diaphysis was not altered by PWB (Supplementary Table 1).

### Quantitative histomorphometry

**Fig. 3:**
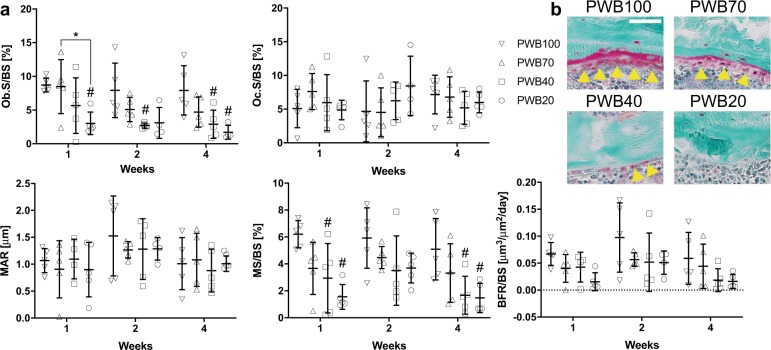
Quantitative trabecular histomorphometry results. Partial weight bearing leads to reductions in osteoblast surface and mineralizing surface **a**. Representative Goldner’s Trichrome image of trabecular bone from 4-week PWB groups **b**. Yellow triangle: osteoblast surface and osteoid; Ob.S/BS osteoblast surface/bone surface, Oc.S/BS osteoclast surface/bone surface, MAR mineral apposition rate, MS/BS mineralizing surface/bone surface, BFR/BS bone formation rate/bone surface; scale bar = 50 μm; Data were analyzed by two-way ANOVA followed by Tukey’s post hoc tests. Data represent mean ± SD. ^#^*p* < 0.05 vs PWB100 within the same time point; *p < 0.05 vs other groups within the same time point.

Deterioration of trabecular microarchitecture was due in part to a decrease in osteoblast surface, which was 65% lower in PWB20 compared to PWB100 after 1 week of unloading. After 4 weeks of unloading, osteoblast surface of both PWB20 and PWB40 were 78% and 63% lower than PWB100, respectively. Osteoclast surface was unaffected by PWB (Fig. 3).

Consistent with the decrease in osteoblast surface, mineralizing surface was decreased in the PWB groups. At 1 week, mineralizing surface was 75% and 40% lower in PWB20 and PWB40, respectively, compared to PWB100. This pattern was maintained with increased unloading time, such that mineralizing surface was 71% and 67% lower in the PWB20 and PWB40 groups, respectively, compared to PWB100 after 4 weeks of unloading. PWB did not alter mineral apposition rate (MAR), though bone formation rate (BFR) tended to be lower, with PWB20 being significantly lower than PWB100 at the 4-week time point.

Unlike alterations in remodeling activity at the trabecular bone, dynamic histomorphometric outcomes were not altered by PWB in cortical bone (Supplementary Table 1). Interestingly, however, periosteal fluorochrome labels were absent in rats that underwent PWB20 for 4 weeks, and may indicate inhibition of periosteal bone formation with longer duration unloading. Accordingly, Ps.MAR, Ps.MS/BS, or Ps.BFR/BS could not be calculated for these animals.

### Femoral biomechanics

**Fig. 4:**
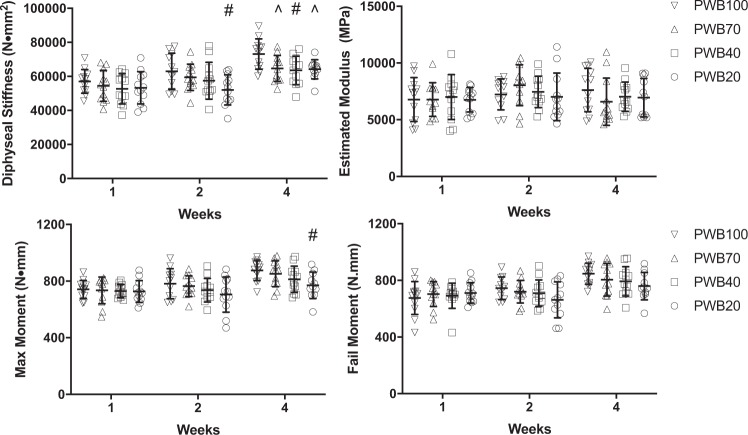
Functional assessment of femoral cortical bone. Partial weight bearing leads to deterioration of diaphyseal stiffness and max moment, but no change in estimated modulus or fail moment. Data were analyzed by two-way ANOVA followed by Tukey’s post hoc tests. Data represent mean ± SD. ^#^*p* < 0.05 vs PWB100 within the same time point; ^*p* < 0.1 vs PWB100 within the same time point.

Three-point bending of the femoral diaphysis revealed modest or no deterioration of functional properties due to PWB (Fig. 4). At 2 weeks, diaphyseal stiffness in PWB20 was lower by 17.4% compared to PWB100. At 4 weeks, all PWB groups exhibited lower stiffness; 11.6% in PWB70; 13.2% in PWB40; and 12.1% in PWB20. Estimated modulus was not altered by PWB.

## Discussion

We hypothesized that partial reductions in mechanical loading would lead to proportional deteriorations in bone mass and structure in rats, and that deficits would be progressive with unloading duration. We tested this hypothesis by examining changes in bone density, microarchitecture, and strength following exposure to varied PWB levels for durations ranging from 1 to 4 weeks. Our results demonstrate dose-dependent and progressive reductions in trabecular vBMD due to PWB, as well as deterioration of trabecular, but not cortical bone microarchitecture.

The strength of current study is assessing skeletal changes induced by PWB at multiple time points. In particular, in vivo longitudinal assessment of vBMD by peripheral quantitative computed tomography (pQCT) overcomes the limitations of cross-sectional ex vivo outcomes, such as microCT and mechanical testing. Increased biological variability in outbred rat strains, which were used in our study, further highlights the advantage of in vivo longitudinal assessment of skeletal changes following PWB. Accordingly, these in vivo measurements revealed a clear dose-dependent response of trabecular bone density to the degree of unloading. Also, rather than reaching a plateau, all PWB regimens led to progressive bone loss for the 4-week duration of the study. Few animal studies have performed long-duration unloading, thus whether bone loss reaches a plateau remains an open question. The progressive nature of the bone loss raises concerns for long-duration spaceflight in the absence of successful bone-sparing countermeasures. The oral bisphosphonate alendronate attenuated the decline in BMD, but potential side effects, such as gastrointestinal intolerance need to be considered^20,21^. Also, advanced resistance exercise device mitigated bone loss, but serum markers for bone resorption continued to be elevated^22^. NASA is planning longer duration missions (perhaps up to 3 years) in the next decades, yet we currently have limited data for skeletal responses to spaceflight durations greater than ~6 months, as well as safe and effective countermeasures. An extreme case of disuse is seen following spinal cord injury. In these patients, bone loss is rapid and extensive, but seems to plateau at ~50% bone loss 2–3 years after the injury^5,23^.

In contrast to observations for the trabecular compartment, cortical bone experienced moderate or no deterioration from PWB in these young adult rats. While cortical BMD measured by pQCT was significantly increased transiently, the increase was modest (~1%) compared to changes in trabecular BMD. The relatively even spread of cortical BMD compared to trabecular BMD among different PWB groups suggests the stability of cortical bone outcomes following PWB. Our prior studies in female C57Bl/6J mice also showed robust dose-dependent bone loss in the trabecular compartment, while the deterioration in the cortical compartment was similar among different PWB groups^15^. The greater response in trabecular bone is likely due to its greater surface area than cortical bone^24^, which allows more basic multicellular units to occur, which then leads to more rapid and robust responses to altered mechanical loading environments^25,26^. Prior studies in the hindlimb suspension model also show greater loss of trabecular bone compared to cortical bone in rats^27^. Another explanation is that to observe tissue and functional level alterations in cortical bone, longer duration of PWB may be needed. The lack of periosteal surface fluorochrome labels in only 4-week PWB20 animals suggests that alterations in cortical bone remodeling may only occur following longer duration exposure to PWB, unlike the trabecular bone that was altered as early as 1-week PWB. Disruption in periosteal cortical bone expansion will likely lead to decrease in the total and cortical bone area, moment of inertia, and ultimately decrease in biomechanical properties.

Data from histomophometry suggest that the deterioration of trabecular bone subsequent to PWB appears to be driven by an imbalance in trabecular bone remodeling, evidenced by a decrease in osteoblast activity with no changes in osteoclast activity. This observation is consistent with tail suspension studies in rodents that report decreased osteoblast activity following unloading^28,29^. Decrease in osteoblast activity seen in PWB groups is likely due to increased sclerostin expression in osteocytes, which has been observed in unloading studies^30,31^. Other studies have shown that hindlimb unloading also increases osteoclast activity, secondary to osteocyte apoptosis^32^, though we did not observe increase in the number of osteoclasts. While this may suggest that even a slight loading by PWB is sufficient to prevent increased osteoclast activity typically associated with unloading in rodent skeleton, further studies are needed to determine whether the cell and molecular mechanisms governing the response to PWB are similar to those reported for other models of unloading. A recent study demonstrated that disuse-associated skeletal muscle transcriptomic profile in mice differs between cast immobilization vs microgravity in the International Space Station, suggesting that different modes of PWB/unloading may also deteriorate skeleton via distinct mechanisms^33^. It remains unclear whether similar mechanisms are involved in the response to skeletal overloading and unloading^34^.

In addition to determining possible mechanisms that may respond differentially to varying degrees of unloading, future work is needed to address the current study’s limitations using a single sex and age of rats. Notably, BFRs were higher in female than male mice after 2 weeks of tail suspension^35^. Also, 2 weeks of unloading by tail suspension led to larger deficits in both the trabecular and cortical compartments in male than in female rats^36^. These studies suggest that female rats may perhaps be more resistant to unloading-induced bone loss than males, though this needs to be tested in the PWB model. Also, aging rats have been reported to respond less to unloading-induced bone loss^37,38^, suggesting that aged rats may be less responsive to PWB-induced bone loss. Despite these limitations, using outbred Wistar rats for our study added strength as the genetic variation is related to human population^39–41^.

In conclusion, this study demonstrates dose-dependent trabecular bone loss in rats, following exposure to differing levels of quadrupedal PWB. The skeletal deficits appear progressive, not reaching a plateau by 4 weeks of PWB. Further elucidating mechanisms underpinning this dose response may lead to the discovery of novel mechanostat^42^ genes that can respond rapidly to alterations in external mechanical unloading environment.

## Methods

### Animals

Male Wistar rats (Charles River Laboratories Inc., Wilmington, MA, USA) weighing 408 ± 0.15 g (14 weeks of age) were obtained and housed in a temperature-controlled (22 ± 2 °C) room with a 12:12-h light–dark cycle. Water and chow were provided ad libitum. Throughout the experiment, rats were assessed daily for any signs of pain or discomfort (e.g., porphyrin staining around eyes, poor grooming, and/or hair loss), harness and jacket fitting, ability to walk and move across the cage, as well as food and water intake. At the end of the study, rats were euthanized by CO_2_ inhalation and hind limbs were harvested. After removing the surrounding soft tissue, the left femur was wrapped in saline-soaked gauze and stored at −20 °C for ex vivo microCT analysis and biomechanical testing, whereas the right femur was stored in 70% ethanol at 4 °C for dynamic histomorphometry. All experimental protocols were approved by the Beth Israel Deaconess Medical Center Institutional Animal Care and Use Committee (IACUC) and animal experiments were conducted according to the guidelines of the IACUC.

### In vivo PWB

Animals were assigned to one of four groups: normal loading (PWB100), and 70% (PWB70), 40% (PWB40), and 20% (PWB20) of normal loading via quadrupedal suspension. These PWB groups were chosen to mimic gravitational forces on Earth (1 G), moderate artificial gravity (0.7 G), Mars (0.4 G), and Moon (0.2 G). Animals were assigned to experimental groups based on pre-PWB body weight to ensure similar average and variability in body weight across all groups. Groups were suspended for 1, 2, or 4 weeks (*n* = 11–12/group). Rats were assigned to each group to ensure balanced distribution of the body weight across all groups. After 2 days of acclimation in custom cages and jackets, rats were suspended according to their PWB groups. Rats were secured in a tether jacket and a pelvic harness and submitted to a two-point suspension allowing them to adopt a normal quadrupedal behavior, while weighing 100, 70, 40, or 20% of their body weight^16^. Animals were weighed daily in their PWB apparatus and the chain link to the two-point suspension was adjusted if needed to ensure that their achieved unloading level remained constant based on PWB groups.

### In vivo trabecular and cortical bone density measurement

At baseline and weekly thereafter, we assessed trabecular vBMD at the right proximal tibia and cortical vBMD at the tibial mid-diaphysis, using in vivo pQCT (Stratec, XCT Research SA+, Pforzheim, Germany). Scans were performed using a voxel size of 100 μm and a scanning beam thickness of 500 μm. Daily calibration was performed using a manufacturer-supplied hydroxyapatite phantom. Transverse images of right tibia were acquired at 5.0, 5.5, 6.0 mm (for total and trabecular vBMD), and at 20 and 20.5 mm (for cortical vBMD) from the proximal tibial plateau. During the scan, rats were under isoflurane-induced anesthesia.

Longitudinal images were visually inspected, and matched based on the distance from the proximal tibial plateau and shape of the proximal tibial metaphysis and fibula to ensure anatomically consistent location for analysis. Scanned slices were analyzed using Stratec software (Stratec XCT Analysis System, v6.0, Norland Corp., Fort Atkinson, WI), using a standardized analysis to determine cortical (contour mode 1, peel mode 2, outer and inner threshold of 0.650 g/cm^3^) and trabecular (contour mode 3, peel mode 4, outer threshold of 0.450 g/cm^3^, inner threshold of 0.800 g/cm^3^) vBMD^16,43^.

### Ex vivo trabecular and cortical bone analyses

Microcomputed tomographic (µCT) imaging was performed on the distal metaphysis and mid-diaphysis of the femur, using a high-resolution desktop imaging system (µCT40, Scanco Medical AG, Brüttisellen, Switzerland) in accordance with the published guidelines for the use of µCT in rodents^44^. Scans were acquired using a 15 µm^3^ isotropic voxel size, 70 kVp and 114 mA peak x-ray tube potential and intensity, 300 ms integration time, and were subjected to Gaussian filtration. We determined femoral length by measuring the distance from the femoral head to the distal end of femoral condyles on µCT scans. The distal metaphyseal region that we analyzed began 600 µm proximal to the distal growth plate and extended proximally 2.1 mm. Cortical bone morphology was evaluated in the femoral mid-diaphysis in a region that started 55% of the bone length below the femoral head and extended 1050 µm distally. Global thresholds of 360 and 700 mg HA/cm^3^ were used for evaluation of trabecular and cortical bone, respectively. Trabecular bone outcomes included trabecular bone volume fraction (Tb.BV/TV, mm^3^/mm^3^), trabecular thickness (Tb.Th, mm), trabecular number (Tb.N, 1/mm), and separation (mm). Cortical bone outcomes included cortical tissue mineral density (Ct.TMD, mg HA/cm^3^), cortical thickness (Ct.Th, µm), total cross-sectional and cortical bone areas (Tt.Ar, Ct.Ar, mm^2^), cortical bone area fraction (Ct.Ar/Tt.Ar, %), and the maximum and minimum moments of inertia (*I*_max_ and *I*_min_, mm^4^).

### Femoral stiffness

The frozen left femurs were thawed and subjected to three-point bending (Instron 8511 MTS, Instron, Norwood, MA). To determine the femoral stiffness (N·mm^2^), maximum moment (N·mm), and failure moment (N·mm), each femur was loaded to failure on the posterior surface at a constant displacement rate of 3 mm/min with the two lower supports spaced 20 mm apart. Force-displacement data were acquired at 100 Hz. Estimated elastic modulus (MPa) was calculated using the moment of inertia derived from microCT scans^45^.

### Dynamic histomorphometry

To examine BFR, calcein (25 mg/kg) was injected intraperitoneally at 10 and 3 days prior to sacrifice. Longitudinal 5-μm thick undecalcified plastic sections were used to measure static and dynamic parameters in trabecular bone in the distal metaphyseal femur, using OsteoMeasure software (Osteometrics; *n* = 5/group). For static histomorphometry, osteoblast (Ob.S/BS) and osteoclast surface (Oc.S/BS) along the trabecular bone was measured. For dynamic histomorphometry, mineralizing surface per bone surface (MS/BS, %) and MAR (μm/day) were measured on unstained sections to calculate BFR (μm^3^/μm^2^/day) according to ASBMR guidelines^46^.

### Statistical analysis

Data were analyzed with GraphPad Prism (GraphPad Software, La Jolla, CA) and SPSS (SPSS Inc., Chicago, IL). To assess changes in in vivo trabecular vBMD measured by pQCT, we used a linear mixed model (fixed variable: PWB, experimental duration, and their interaction; repeated variable: experimental duration) followed by pairwise comparisons with Bonferroni correction. Ex vivo outcomes were analyzed by two-way ANOVA followed by Tukey’s post hoc tests. To examine the dose-dependent nature of the response to PWB, we performed a test for trend^47^. Results are presented as mean ± SEM and were considered significant when *p* < 0.05.

## Supplementary information

supplementary-materials

reporting-summary

## Data Availability

The data that support the findings of this study area available from the corresponding author upon reasonable request.
