# Supplementary material for: Dose-dependent skeletal deficits due to varied reductions in mechanical loading in rats
Source: NPJ Microgravity. 2020 May 18;6:15. doi: 10.1038/s41526-020-0105-0 (PMC7235020; doi:10.1038/s41526-020-0105-0)
Supplement: Supplementary file 1 — supplementary-materials [file 41526_2020_105_MOESM1_ESM.pdf]

Supplementary Table 1. MicroCT and dynamic histomorphometry outcome of cortical bone parameters (mean ± SD)

	**1-week**				**2-week**				**4-week**				***P* value**		
	**PWB100**	**PWB70**	**PWB40**	**PWB20**	**PWB100**	**PWB70**	**PWB40**	**PWB20**	**PWB100**	**PWB70**	**PWB40**	**PWB20**	**PWB**	**Duration**	**Interaction**
**Ct.TMD [mg HA/cm^3]**	1095 ± 6	1102 ± 6	1102 ± 6	1097 ± 4	1101 ± 5	1113 ± 4	1109 ± 5	1095 ± 5	1112 ± 4	1106 ± 4	1109 ± 6	1102 ± 6	0.08	0.14	0.62
**Ct.Th [µm]**	620 ± 14	623 ± 17	624 ± 14	615 ± 9	629 ± 12	643 ± 14	633 ± 11	616 ± 10	648 ± 14	636 ± 10	649 ± 13	630 ± 13	0.48	0.08	0.95
**Ct.Ar [mm^2]**	6.60 ± 0.18	6.46 ± 0.15	6.39 ± 0.12	6.34 ± 0.08	6.56 ± 0.13	6.39 ± 0.14	6.35 ± 0.14	6.29 ± 0.15	7.00 ± 0.14	7.05 ± 0.19	6.84 ± 0.17	6.88 ± 0.22	0.29	<0.0001	0.99
**Tt.Ar [mm^2]**	11.6 ± 0.49	11.2 ± 0.29	10.9 ± 0.35	10.9 ± 0.28	11.7 ± 0.49	10.7 ± 0.35	10.9 ± 0.31	10.8 ± 0.36	12.4 ± 0.57	12.7 ± 0.55	11.9 ± 0.46	12.3 ± 0.53	0.23	<0.0001	0.78
**Ct.Ar/ Tt.Ar [%]**	57.2 ± 1.4	58.1 ± 1.4	59.2 ± 1.4	58.2 ± 1.2	56.6 ± 1.5	60.2 ± 1.3	58.6 ± 0.9	58.5 ± 1.0	57.5 ± 1.6	56.2 ± 1.5	58.1 ± 1.1	56.1 ± 1.4	0.52	0.25	0.64
**I_{max} [mm^4]**	11.5 ± 0.9	10.2 ± 0.4	10.0 ± 0.5	10.2 ± 0.4	11.4 ± 0.9	9.5 ± 0.5	9.6 ± 0.5	9.8 ± 0.6	13.1 ± 1.0	13.0 ± 0.9	11.8 ± 0.9	12.8 ± 0.9	0.30	<0.0001	0.66
**I_{min} [mm^4]**	6.37 ± 0.47	6.14 ± 0.31	5.74 ± 0.31	5.70 ± 0.26	6.54 ± 0.40	5.79 ± 0.34	5.99 ± 0.34	5.64 ± 0.34	7.17 ± 0.57	7.96 ± 0.59	6.92 ± 0.45	7.15 ± 0.60	0.06	<0.0001	0.91
**En.MAR [µm]**	2.11 ± 0.19	2.19 ± 0.36	1.91 ± 0.18	2.51 ± 0.32	2.95 ± 0.27	2.89 ± 0.37	3.12 ± 0.58	2.91 ± 0.63	2.39 ± 0.18	2.06 ± 0.14	2.91 ± 0.63	3.09 ± 0.61	0.54	0.03	0.77
**En.MS/BS [%]**	30.3 ± 4.9	30.2 ± 8.4	40.0 ± 7.6	36.1 ± 2.6	34.6 ± 5.3	33.4 ± 5.0	28.6 ± 5.2	42.3 ± 10	36.2 ± 7.3	22.5 ± 4.9	44.5 ± 15	41.3 ± 5.2	0.29	0.93	0.65
**En.BFR/BS [µm^3/µm^2/day]**	0.63 ± 0.12	0.77 ± 0.30	0.82 ± 0.19	0.93 ± 0.16	1.02 ± 0.18	1.02 ± 0.26	0.97 ± 0.29	1.41 ± 0.63	0.90 ± 0.24	0.48 ± 0.13	1.57 ± 0.74	1.31 ± 0.38	0.32	0.36	0.70
**Ps.MAR [µm]**	1.49 ± 0.07	1.68 ± 0.13	1.89 ± 0.09	1.78 ± 0.15	2.43 ± 0.37	2.04 ± 0.17	2.49 ± 0.71	1.97 ± 0.12	1.63 ± 0.41	1.45 ± 0.26	2.07 ± 0.22	*	0.89	0.03	0.85
**Ps.MS/BS [%]**	21.8 ± 5.9	22.6 ± 4.7	22.4 ± 7.8	25.4 ± 3.4	35.1 ± 8.0	25.2 ± 6.9	19.8 ± 2.6	20.1 ± 9.0	25.2 ± 7.7	13.2 ± 1.7	8.48 ± 2.8	*	0.27	0.20	0.63
**Ps.BFR/BS [µm^3/µm^2/day]**	0.40 ± 0.09	0.38 ± 0.10	0.53 ± 0.15	0.46 ± 0.10	0.94 ± 0.33	0.52 ± 0.17	0.54 ± 0.16	0.40 ± 0.18	0.50 ± 0.24	0.21 ± 0.07	0.23 ± 0.03	*	0.27	0.09	0.53
